# Synergistic effects of curcumin and bevacizumab on cell signaling pathways in hepatocellular carcinoma

**DOI:** 10.3892/ol.2014.2694

**Published:** 2014-11-10

**Authors:** JIAN-ZHI GAO, JING-LI DU, YONG-LING WANG, JIA LI, LI-XIN WEI, MING-ZHOU GUO

**Affiliations:** 1 Department of Pathology, General Hospital of the People’s Liberation Army, Beijing 100853, P.R. China; 2 Basic Medical College of Xinxiang Medical University, Xinxiang, Henan 453003, P.R. China; 3 Department of Gastroenterology and Hepatology, General Hospital of the People’s Liberation Army, Beijing 100853, P.R. China; 4 Department of Gastroenterology, Armed Police Corps Hospital of Qinghai, Xining, Qinghai 810006, P.R. China

**Keywords:** curcumin, vascular endothelial growth factor blocker, signaling pathway, vascular endothelial growth factor, K-ras, hepatocellular carcinoma

## Abstract

The aim of the present study was to explore the effects of curcumin in combination with bevacizumab on the vascular endothelial growth factor (VEGF)/VEGF receptor (VEGFR)/K-ras pathway in hepatocellular carcinoma. A total of 30 Sprague Dawley (SD) rats were randomly divided into five groups: Control, model, curcumin, VEGF blocker, and curcumin + VEGF blocker groups. The mRNA levels of VEGF and VEGFR in all groups were subsequently measured by quantitative reverse transcriptase-polymerase chain reaction and the protein expression of K-ras was detected by western blot analysis. Compared with the control group, the mRNA levels of VEGF and VEGFR were revealed to be significantly increased in the model, curcumin and VEGF blocker groups. The VEGF mRNA levels in the curcumin, VEGF blocker and curcumin + VEGF blocker groups were all decreased when compared with the model group. In addition, the VEGF mRNA levels in the curcumin + VEGF blocker group were significantly lower compared with the curcumin group (P<0.05). The VEGF mRNA levels in the curcumin, VEGF blocker and curcumin + VEGF blocker groups were decreased when compared with the model group (P=0.0001). No significant differences in VEGF mRNA levels were identified between the VEGF blocker and curcumin groups (P=0.863), whereas the VEGF mRNA levels in the curcumin + VEGF blocker group were significantly lower than that of the curcumin group (P=0.025). Curcumin and the VEGF blocker are each capable of inhibiting hepatocellular carcinoma progression by regulating the VEGF/VEGFR/K-ras pathway. The combination of the two compounds has a synergistic effect on the inhibition of the effects of the VEGF signaling pathways in hepatocellular carcinoma progression.

## Introduction

Curcumin is a polyphenolic compound that is extracted from ginger, turmeric and *Curcuma* rhizomes and exerts a wide range of antitumor effects, including the induction of tumor cell apoptosis, cell cycle arrest, and exhibits anti-invasion and anti-angiogenic properties (1). Previous studies have revealed that the growth, invasion and metastasis of tumors all depend on angiogenesis (2–4). Vascular endothelial growth factor (VEGF), comprising the superfamily of VEGFs, VEGF receptors (VEGFRs), downstream signaling proteins and certain nuclear transcription factors, can stimulate the proliferation of endothelial cells, which indicates an association between VEGF and the development, invasion and metastasis of tumors (5). Previous studies have also revealed that VEGF is significant in hepatocellular carcinoma (HCC) progression (6–10). Bevacizumab, a VEGF blocker, is a humanized monoclonal antibody for VEGF that implements its antitumor effects by inhibiting VEGF signaling pathways (10,11). In the present study, a rat HCC model is employed to determine the changes in mRNA levels of VEGF and VEGFR, and the expression of the K-ras protein when curcumin is administered together with a biological target, in order to provide experimental evidence for design comprehensive therapy and improve clinical outcomes.

## Materials and methods

### Materials

The diethylnitrosamine (DENA) and curcumin were purchased from Sigma-Aldrich (St. Louis, MO, USA). Bevacizumab (100 mg/4 ml) was purchased from People’s Liberation Army General Hospital (Beijing, China). K-ras mouse monoclonal antibodies were purchased from Santa Cruz (Dallas, TX, USA; sc-30), and GAPDH mouse anti-rat monoclonal antibodies (TDY042) and the western blot detection kit were purchased from Tian De Yue Biological Technology Company (Beijing, China). The 30 male SD rats, which weighed between 150 and 180 g, were provided by the Experimental Animal Center of Henan Province (Henan, China).

### Establishment of the rat hepatoma model

**Figure 1:**
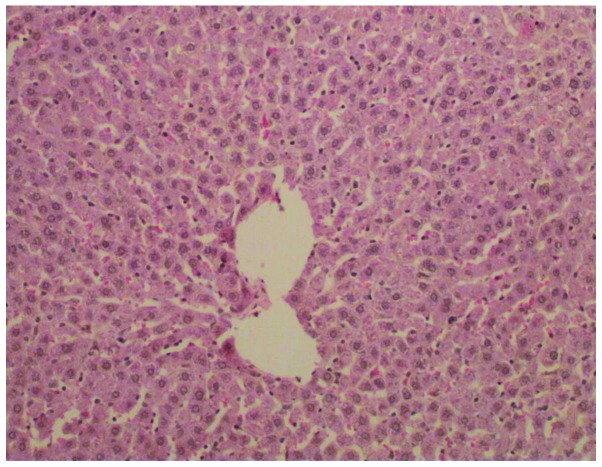
Hematoxylin and eosin staining of tissue from the normal control group (magnification, ×200).

**Figure 2:**
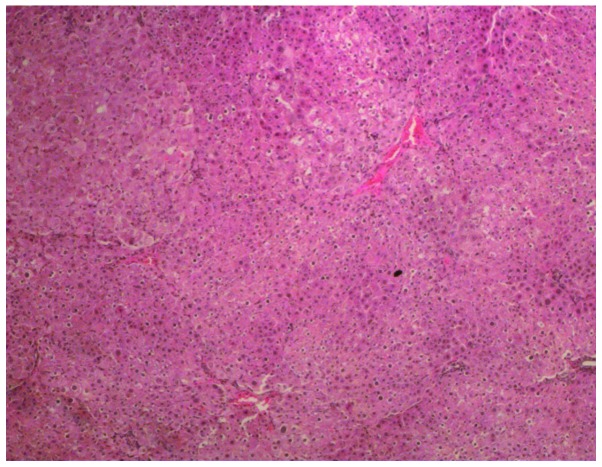
Hematoxylin and eosin staining of tissue from the hepatocellular carcinoma model group (magnification, ×200).

According to the method described by Futakuchi *et al* (12), the 30 SD rats were randomly divided into five groups, with six rats in each group, which were termed the control (Fig. 1), model (Fig. 2), curcumin, VEGF blocker and curcumin + VEGF blocker groups. All the groups, with the exception of the control group, were intragastrically administered once per week with 70 mg/kg of DENA for eight weeks. The model group was continuously intragastrically administered with DENA. The curcumin group was continuously intragastrically administered with DENA and curcumin solution, which consisted of curcumin dissolved in a 0.5% sodium carboxymethyl cellulose solution to yield a 2% concentration of curcumin suspension that was administered at 20 mg/kg of body weight every second week for six weeks. The VEGF blocker group was intragastrically administered with DENA and 0.5% sodium carboxymethyl cellulose between weeks 9–18, and with 0.003 ml/g bevacizumab by intraperitoneal injection every second week for six weeks. The curcumin + VEGF blocker group was administered with DENA and curcumin from the beginning of week nine, followed by intraperitoneal injection of bevacizumab from week 10, as with the VEGF blocker group. The control group was administered with saline for the first eight weeks, followed by saline plus 0.5% sodium carboxymethyl cellulose by gavage between weeks 9–18. All animals were sacrificed at week 18, the liver tissue was dissected from the control group and a small hepatocellular carcinoma was dissected from each rat in the other four groups. A portion of the tissue was placed in 4% paraformaldehyde for fixture, and the remainder was frozen in liquid nitrogen.

### Western blot analysis of K-ras protein

All samples were segmented and lysed in protein lysis buffer overnight at 4°C and the protein concentrations were then quantified using the coomassie blue method. An equal amount of each protein sample was subjected to polyacrylamide gel electrophoresis, transferred onto a polyvinylidene difluoride membrane, blocked with milk for 4 h and incubated with monoclonal mouse anti-rat K-ras primary antibody (1:200; sc-30; Santa Cruz) overnight at 4°C. The samples were subsequently incubated with polyclonal goat anti-mouse secondary antibodies (S001; Tian De Yue Biological Technology Company, Beijing, China) for 2 h at room temperature and chemiluminescence was detected using an electrochemiluminescence western blot detection kit (Pierce Biotechnology, Inc., Rockford, IL, USA). The western blot band density was analyzed using ImageJ software version 1.57 (National Institutes of Health, Bethesda, MD, USA).

### Total RNA extraction and reverse transcriptase-polymerase chain reaction (RT-PCR)

All the primers were designed according to the cDNA sequences in GenBank (National Center for Biotechnology Information, Bethesda, MD, USA), and were synthesized at the Beijing Sainuobo Biotechnology Center (Beijing, China). The PCR amplification for VEGF and VEGFR was performed initially at 95°C for 10 min, and then at 95°C for 15 sec followed by 60°C for 60 sec, for 45 cycles. The primer sequences used in PCR were as follows: VEGFR forward, 5′-ACGGACAGTGGTATGGTTCTTGCC-3′ and reverse, 5′-GGTAGCCGCTTGTCTGGTTTGAG-3′ (amplified fragment length, 145 bp); VEGF forward, 5′-CCACTGAGGAGTCCAACATCACCAT-3′ and reverse, 5′-CGGGATTTCTTGCGCTTTCGT-3′ (amplified fragment length, 190 bp); and internal control β-actin (ACTB) forward, 5′-ACTTAGTTGCGTTACACCCTT-3′ and reverse, 5′-GTCACCTTCACCGTTCCA-3′ (amplified fragment length, 156 bp). All the mRNA levels were normalized against ACTB and were expressed as relative values using the 2^−ΔΔCT^ method: ΔΔCT = (Ct_target gene_ − Ct_ACTB_)_HCC_ − (Ct_target gene_ − Ct_ACTB_)_control liver tissue_.

### Statistical analysis

All the data are presented as the mean ± standard error of the mean. SPSS version 13.0 statistical software (SPSS, Inc., Chicago, IL, USA) was used for analysis of variance. The correlation between the VEGFR mRNA level and K-ras protein expression was analyzed using Pearson correlation. P<0.05 was considered to indicate a statistically significant difference.

### Ethics statement

The present study was approved by the Xinxiang University Animal Care and Use Committee (Xinxiang, Henan, China) and the ethics committee of Xinxiang Medical University. The mice were maintained, bred, animal modeled, sacrificed and utilized in accordance with Xinxiang University Animal Care and Use Committee.

## Results

### The mRNA levels of VEGF and VEGFR in HCC groups

Compared with the control group, the mRNA levels of VEGF and VEGFR were revealed to be significantly increased in the model, curcumin and VEGF blocker groups (P<0.05), but not in the curcumin + VEGF blocker group (P>0.05). The VEGF mRNA levels in the curcumin, VEGF blocker and curcumin + VEGF blocker groups were all lower compared with the model group (P<0.05). No significant difference was identified between the VEGF mRNA levels in the VEGF blocker group and those in the curcumin group (P>0.05), whereas those in the curcumin + VEGF blocker group were lower than those in the curcumin group (P<0.05). In addition, the VEGF mRNA levels in the curcumin + VEGF blocker group were significantly lower compared with the VEGF blocker group (P<0.05). The relative VEGFR mRNA levels in the curcumin group and curcumin + VEGF blocker group were significantly lower compared with the model group (P<0.05), however, no significant difference was identified between the VEGF blocker group and model group (P>0.05), or between those in the VEGF blocker and curcumin + VEGF blocker group and in the curcumin group (P>0.05). The VEGFR mRNA levels in the curcumin + VEGF blocker group were significantly lower compared with the VEGF blocker group (P<0.05) (Table I).

### K-ras protein expressions in HCC groups

**Figure 3:**
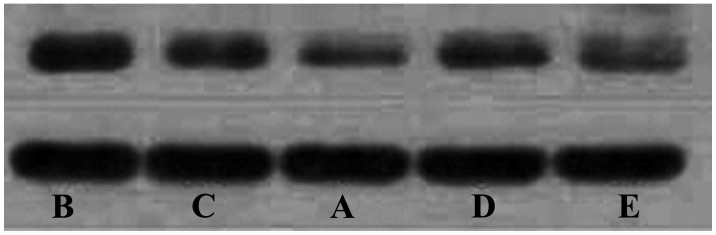
The relative expression of K-ras protein in the (A) control, (B) model, (C) curcumin, (D) VEGF blocker and (E) curcumin + VEGF blocker groups. VEGF, vascular endothelial growth factor.

The protein expression of K-ras was significantly lower in the curcumin, VEGF blocker and curcumin + VEGF blocker groups compared with the model group (P<0.05). The protein expression of K-ras in the VEGF blocker and curcumin groups were similar (P>0.05), and the two levels were significantly higher compared with curcumin + VEGF blocker group (P<0.05) (Fig. 3; Table II).

### Correlation between VEGF/VEGFR mRNA and K-ras protein

Pearson correlation analysis revealed that the VEGFR mRNA and K-ras protein in all HCCs exhibit a significant positive correlation (r=0.835; P=0.0001).

## Discussion

HCC has a high incidence worldwide and is the third most prevalent cause of cancer-related mortality (13). HCC is highly vascular and the progression, invasion and metastasis of HCC all depend on angiogenesis. VEGF is a vascular endothelial, cell-specific, heparin-binding growth factor that is capable of inducing angiogenesis. VEGF is one of the predominant factors that induces angiogenesis in tumors by means of paracrine and autocrine signaling; this results in tumor growth and metastasis. The induction of VEGF in HCC in the present study was consistent with that observed in previous studies (5,10).

At present, inhibiting tumor angiogenesis and blocking the corresponding signaling pathway has become the focus of numerous studies investigating basic medical and clinical research in cancer treatment (14,15). As VEGF is significant in HCC progression, it may be of therapeutic benefit in the downregulation of VEGF/VEGFR/K-ras signaling pathways. The oncogene Ras is a member of the oncogene family, consisting of H-ras, K-ras and N-ras (16). Numerous studies have revealed that a loss of control over oncogenes and tumor suppressor genes is closely associated with tumor progression, invasion and metastasis (17–19).

In recent years, studies have revealed that a variety of other risk factors, including chronic alcoholism and hepatitis B viral infection, may induce the increased expression of growth factors, including VEGF and PDGF-β, and their receptors in the cytoplasm and membrane of hepatocytes (20–22). The binding of induced growth factors with the corresponding receptors leads to the activation of receptor tyrosine kinase signaling pathways and hyperphosphorylation at the carboxyl terminus of receptor tyrosine kinase, which results in the hyperactivation of Ras protein kinases (23–25). Activated Ras, through its downstream signaling pathway, inhibits apoptosis and promotes cell proliferation and survival, thus eventually leading to abnormal cell proliferation and tumor formation (26).

Bevacizumab is a recombinant humanized anti-VEGF monoclonal antibody that was developed by Roche (Basel, Switzerland). The half-life in humans is 17–21 days, and humanization can extend its half-life. Bevacizumab, which consists of 7% rat structure and 93% human IgG fragments, targets VEGF and competitively binds the VEGF receptor, resulting in a blockage of VEGF-mediated downstream signaling pathways and the inhibition of VEGF-induced vascular endothelial cell proliferation and tumor angiogenesis. These effects block the blood, oxygen and other nutrients that are necessary for tumor growth, thus exerting its antitumor effect via the limitation of tumor growth (12). Therefore, the inhibition of angiogenesis in tumors and blockade of the corresponding signaling pathways has become the focus of much basic medical and clinical research investigating cancer treatments.

The present study reveals that compared with the control group, the mRNA levels of VEGF and VEGFR, and the expression of the K-ras protein in the HCC model groups were all significantly increased. In addition, compared with the model group, the mRNA level of VEGF in the VEGF blocker group was significantly reduced, whereas the mRNA level of VEGFR was not reduced; additionally, the expression of the K-ras protein in the HCC tissue was also reduced. All results indicate the significance of VEGF signaling pathways in the progression of HCC, demonstrating that VEGF is a promising target for tumor-targeted therapy.

Curcumin is a turmeric polyphenol extracted from the rhizome of ginger plants; it exerts several important effects, including anti-oxidant and anti-inflammatory effects, lowering of blood pressure and anti-atherosclerotic and antitumor effects (27,28). Previous studies have revealed that curcumin may prevent a variety of cancers, including esophageal, breast and colon cancers (29–31). Curcumin has strong effects on the inhibition of tumor cell growth, characterized by the induction of apoptosis, and the inhibition of angiogenesis in tumor tissue via the promotion of cytochrome C release. Curcumin also regulates the Akt, NF-κB, AP-1 and JNK signaling pathways (32,33). Furthermore, a number of studies have reported that curcumin may inhibit the abnormal proliferation of liver cancer cells in a time- and concentration-dependent manner (34).

Although the antitumor effect of curcumin has been studied for years, it remains unclear whether or not the VEGF signaling pathway is of significance. One mechanism underlying the antitumor effect of curcumin is the inhibition of tumor angiogenesis (35). However, several questions remain, including whether this effect is due to blocking the VEGF signaling pathway and if so, whether there is any competition or synergistic effects when curcumin is used in combination with bevacizumab. These questions were addressed in the present study.

The results in the current study reveal that when treated with curcumin, rat HCC tissues exhibit lower VEGF and VEGFR mRNA levels compared with the model group. The curcumin group also exhibited lower K-ras protein expression compared with the HCC model group. These observations indicate that curcumin may inhibit HCC progression, invasion and metastasis by decreasing the expression of VEGF, VEGFR and K-ras. Following treatment with bevacizumab in combination with curcumin, the VEGF mRNA level was lower compared with the curcumin and VEGF blocker groups, but was not significantly different from the level observed in the control group. Furthermore, the VEGFR mRNA level was lower compared with the VEGF blocker group, but was not significantly different from the curcumin group. The K-ras protein expression in the HCC tissue of the curcumin + VEGF blocker group was also lower compared with the curcumin and VEGF blocker groups. In addition, the relative expression of VEGFR in each group exhibited a significant positive correlation with the K-ras protein expression. These observations indicate that the combination of the two compounds has a synergistic effect, which provides a theoretical and experimental basis for the comprehensive clinical evaluation in targeted therapy.

In conclusion, these results indicate that curcumin is capable of reducing the expression of VEGF, VEGFR and K-ras, which results in the repression of HCC tumor growth, invasion and metastasis, and that curcumin interacts with the same signaling pathway as bevacizumab. The results from the combination of curcumin with bevacizumab indicate that the two have a synergistic effect rather than a competitive effect, which provides confirmation for the potential benefits of the combined use of anti-HCC drugs that target the same signaling pathway.

## Figures and Tables

**Table I:** 2^−ΔΔct^ of expression of VEGF and VEGFR mRNA in each group (n=6).

Group	VEGF/ACTB	VEGFR/ACTB
Control	0.85±0.17^b^^c^^d^	0.78±0.13^b^^c^^d^
Model	4.96±0.88^a^^c^^d^^e^	4.45±1.18^a^^d^^e^
Curcumin	1.69±0.28^a^^b^^e^	2.28±0.43^a^^b^^d^
VEGF blocker	1.93±0.40^a^^b^^e^	4.08±1.21^a^^c^^e^
Curcumin + VEGF blocker	1.12±0.08^b^^c^^d^	1.93±0.38^b^^c^^d^

Data are presented as the mean ± standard deviation;

a P<0.05 vs. control group;

b P<0.05 vs. model group;

c P<0.05 vs. curcumin group;

d P<0.05 vs. VEGF blocker group;

e P<0.05 vs. curcumin + VEGF blocker group.

VEGF, vascular endothelial growth factor; VEGFR, VEGF receptor; ACTB, β-actin.

**Table II:** The relative expression of K-ras protein in each group (n=6).

Group	K-ras/GADPH
Control	0.54±0.07^b^^c^^d^
Model	0.85±0.12^a^^c^^d^^e^
Curcumin	0.67±0.09^a^^b^^e^
VEGF blocker	0.67±0.06^a^^b^^e^
Curcumin + VEGF blocker	0.56±0.07^a^^b^^c^^d^

Data are presented as the mean ± standard deviation;

a P<0.05 vs. control group;

b P<0.05 vs. model group;

c P<0.05 vs. curcumin group;

d P<0.05 vs. VEGF blocker group;

e P<0.05 vs. curcumin + VEGF blocker group.

VEGF, vascular endothelial growth factor.
